# Enhanced optical confinement and lasing characteristics of individual urchin-like ZnO microstructures prepared by oxidation of metallic Zn

**DOI:** 10.1186/1556-276X-9-178

**Published:** 2014-04-11

**Authors:** Chia-Hao Lu, Tzu-Yang Chao, Ying-Feng Chiu, Shuo-Yen Tseng, Hsu-Cheng Hsu

**Affiliations:** 1 Department of Photonics, National Cheng Kung University, Tainan 70101, Taiwan; 2 Advanced Optoelectronic Technology Center, National Cheng Kung University, Tainan 70101, Taiwan

**Keywords:** Random laser, ZnO, Microstructures

## Abstract

We prepared urchin-like micron-sized ZnO cavities with high optical quality by oxidizing metallic Zn and proposed the mechanism that resulted in the growth of the urchin-like microstructures. The photoluminescence spectra of the ZnO microstructures had a predominant excitonic emission at room temperature. The lasing properties of the urchin-like ZnO microstructures were investigated systematically through excitation power- and size-dependent photoluminescence measurements. The results showed that a low lasing threshold with high quality factors could be achieved because of the high reflectivity of the optical reflectors formed by the tapered nanowires. The unique optical characteristics may facilitate the development of high-efficiency random lasers.

## Background

The miniaturization of light sources is one of the key issues for the development of smaller optoelectronic devices with enhanced functions and properties [1-4]. Zinc oxide (ZnO) materials have attracted increased attention in recent years to realize efficient UV emitters because of their large direct bandgap of 3.37 eV and large free exciton binding energy of 60 meV [5-7]. Remarkable efforts have already been devoted to the synthesis of various ZnO nano/microstructures such as nanowires, nanobelts, nanoribbons, nanorods, and microdisks, which serve as the most promising building blocks for nano/microsized optoelectronic devices [8-16]. UV lasing action at room temperature using ZnO nano/microstructures has significantly spurred the research interest. The lasing characteristics of ZnO micro/nanostructures can generally be classified into two feedback mechanisms: microcavity lasing and random lasing (RL). In the case of microcavity lasing, light confinement is attributed to the high refractive index of ZnO, and the light can be amplified within a single ZnO micro/nanocrystal. There are two ways of confining light: using a Fabry-Pérot (F-P) cavity in a ZnO nanowire [2,8,9] and using a whispering-gallery mode (WGM) cavity in a single ZnO microrod [7,15,17] or microdisk [18]. Because microcavity lasers have a high spatial coherence, the light that emerges from the laser can be focused on a diffraction-limited spot or propagated over a long distance with minimal divergence. On the other hand, RL is caused by light scattering, and random oscillation routes are created by using numerous ZnO micro/nanocrystals or a ZnO microsized composited random medium [10-12,19,20]. The coherence properties of RL have been demonstrated in the literature. According to the photon statistics theory, the photon distribution for a coherent light source obeys a Poisson distribution, and the photon distribution for an incoherent light source follows a Bose-Einstein distribution. The temporal coherence properties of a random laser were investigated by using a Michelson interferometer [21]. Cao et al. [22] studied the photon statistics of a single-shot random laser mode fit to a Poisson-like distribution upon high-intensity pumping. They also addressed the low spatial coherence of RL emission using double-slit experiments [23]. The RL exhibited a high intensity with low spatial coherence due to the stimulated emission in many different spatial modes. Optoelectronic and medical applications require low spatial coherence such as for high-resolution speckle-free imaging. Therefore, it has been conceptually demonstrated that RL is superior to conventional lasing for speckle-free imaging applications [24].

The RL-related effects have been demonstrated in different ZnO architectures. Most previous studies on RL with ZnO architectures have been accomplished on ensembles [10-12,19,20], meaning the properties of the individual microstructures were missing in the superposition of the ensemble. However, the RL characteristics of single microstructures have not been investigated so far. A detailed investigation on the lasing behaviors of the individual ZnO microstructures is crucial for micro/nanolaser application. In this study, we demonstrated a type of urchin-like ZnO microcrystal formed by oxidizing metallic zinc and revealed the excellent optical quality of these ZnO microstructures. Furthermore, the random lasing behavior of a single urchin-like microstructure was comprehensively examined by employing the excitation power and microstructure size dependence of the photoluminescence emission by pulsed laser excitation.

## Methods

The synthesis of ZnO microcavities was conducted in two steps. First, hexagonal Zn microcrystals were fabricated using carbothermal vapor-phase transport [14]. This step involved placing a source that contained ZnO powder and graphite powder at a volume ratio of 1:1 into a furnace tube and then placing a Si (100) substrate in a downstream position. After the system was evacuated to a pressure of less than 100 mTorr using a mechanical pump, high-purity argon gas was introduced into the system at a flow rate of 10 sccm. The temperature was kept at 950°C for 1 h, and the pressure in the tube was maintained at 800 mTorr. Then, we conducted an oxidation process. The pressure inside the furnace tube was maintained at 800 mTorr (the same pressure used in the first step) with an O_2_ flow of 5 sccm, and the oxidation process was conducted at 500°C for 1 h. The synthesized products were characterized by scanning electron microscopy (SEM) and X-ray diffraction (XRD).

For the optical study of a single object, the products were dispersed onto a marked substrate to identify and locate the positions of the individual microcavities. In order to easily locate the excitation area for pumping each individual ZnO microcavity, a 200-mesh transmission electron microscopy grid was fixed on the sample. To measure the photoluminescence, a micro-photoluminescence (μ-PL) system was used to analyze the optical properties of the individual ZnO microcavities under the excitation of a 325-nm HeCd laser or a 266-nm Nd: YAG pulsed laser. The sample was placed on a sample holder that was mounted on a three-axis translational stage. A camera was used to distinguish the signals emitted from individual ZnO microcavities. All of the optical measurements were performed at room temperature.

## Results and discussion

**Figure 1:**
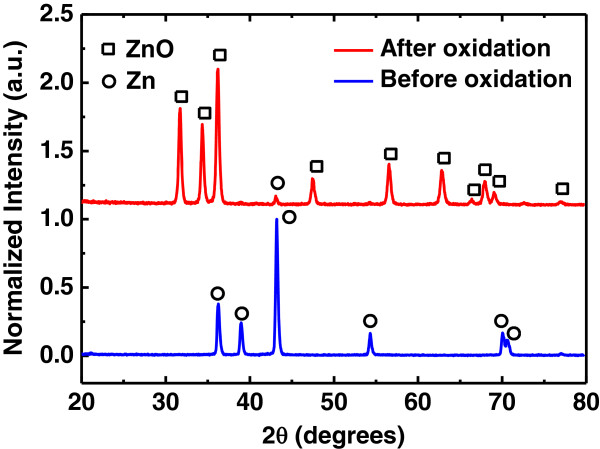
**XRD patterns of the Zn microcrystal (bottom branch) and the annealed sample (upper branch).** The circles denote peaks corresponding to Zn and the squares to ZnO.

Figure 1 shows the typical XRD patterns of the products synthesized in the first and second steps. For the products that were obtained before the oxidation process, all of the peaks were identified as Zn with a hexagonal structure (JCPDS No. 87-0713); no obvious diffraction peaks of ZnO were identified because there was no diffraction pattern attributed to the impurities. After the oxidation process, almost all of the diffraction peaks could be readily indexed as the hexagonal wurtzite ZnO phase (JCPDS No. 36-1451), except for the Zn peak at 43.36°. These results indicated that the Zn crystals were oxidized. The Zn could have originated from the inner core of the first products, where the Zn had yet to be transformed fully into the ZnO structures.

**Figure 2:**
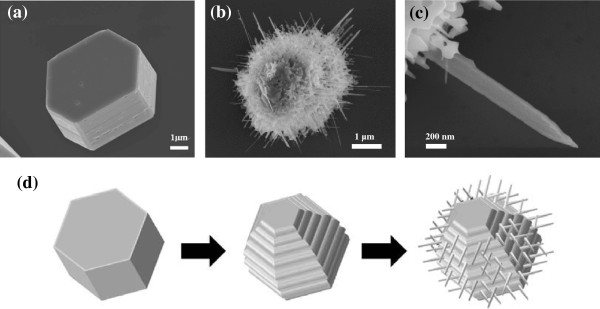
**SEM images of individual ZnO microcrystal, magnification image of tapered nanowire, and the oxidation process.** SEM images of an individual ZnO microcrystal **(a)** before and **(b)** after oxidation at 500°C. **(c)** The magnification image of the tapered nanowire. **(d)** Illustration images of the metallic Zn transformed into ZnO microcavity during the oxidation process.

Figure 2a shows a representative SEM image of the morphology of the product fabricated during the first step. The figure shows hexagonal Zn/ZnO microcrystals with six-faceted side walls. The diameter and height of the Zn/ZnO microcrystals were 4.5 and 1.5 μm, respectively. A low-magnification SEM image of a large area (not shown) showed that these microcrystals had diameters that ranged from 3 to 16 μm. After the oxidation process in step 2, urchin-like ZnO microstructures with multilayer sheets and multiple nanowires were observed, as shown in Figure 2b. Figure 2c shows an enlarged image of the typical nanowire with a tapered structure. The diameters and lengths of the tapered nanowires had ranges of 70 to 300 nm and 0.5 to 10 μm, respectively.

The growth mechanism of these urchin-like structures was proposed to be self-catalyzed growth resulting from the oxidation of metallic Zn. Figure 2d shows the proposed mechanism by which these urchin-like ZnO microstructures were formed. First, graphite served as a reducing agent. Then, Zn vapor was generated through the carbothermal reduction of ZnO powder at high temperature. The Zn vapor was carried to a low-temperature region by the flow of Ar gas, and the result was the condensation of Zn microcrystals onto the Si substrate located downstream. The zinc microcrystals had the morphology of hexagonally shaped platelets. Second, in the O_2_ environment that existed during the oxidation process, the as-grown Zn microcrystals were transformed into sheets with side faces that were flat [14]. The oxidation of Zn was caused by the increased surface mobility of the nanosized, liquid Zn droplets and oxygen atoms, which induced the nucleation and growth of ZnO crystals into nanowires. The side face of each flat plane was covered with armlike nanowire structures, hence the name ‘urchin-like’ microstructures.

**Figure 3:**
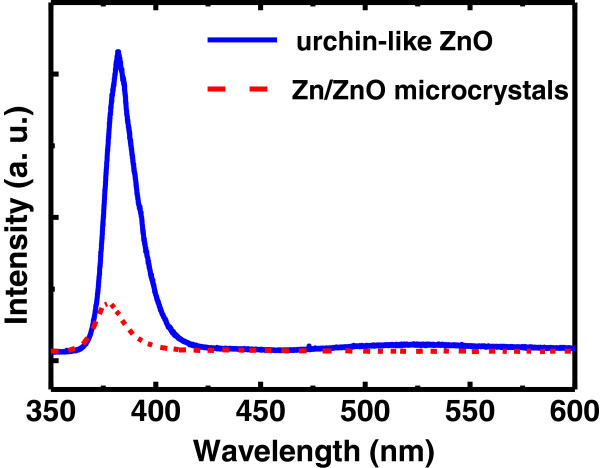
Micro-PL spectra of the sample before and after oxidation by cw-laser excitation.

Figure 3 shows the μ-PL spectra of the Zn/ZnO microcrystals (solid line) and urchin-like ZnO microstructures (dashed line). The PL spectrum of the urchin-like ZnO microstructures shows an excitonic UV emission centered at 382 nm and a relatively weak emission associated with defects located at 522 nm. The intensity of the UV emission is five times greater than that of the as-grown sample. The appearance of the UV emission from these microcrystals indicates that the Zn, which can be oxidized quickly, has been partially oxidized to form a thin ZnO layer on the surface. A blue shift in the UV band can be interpreted by the quantum confined effect to indicate that the thickness of the native oxide on the surface is just a few nanometers.

**Figure 4:**
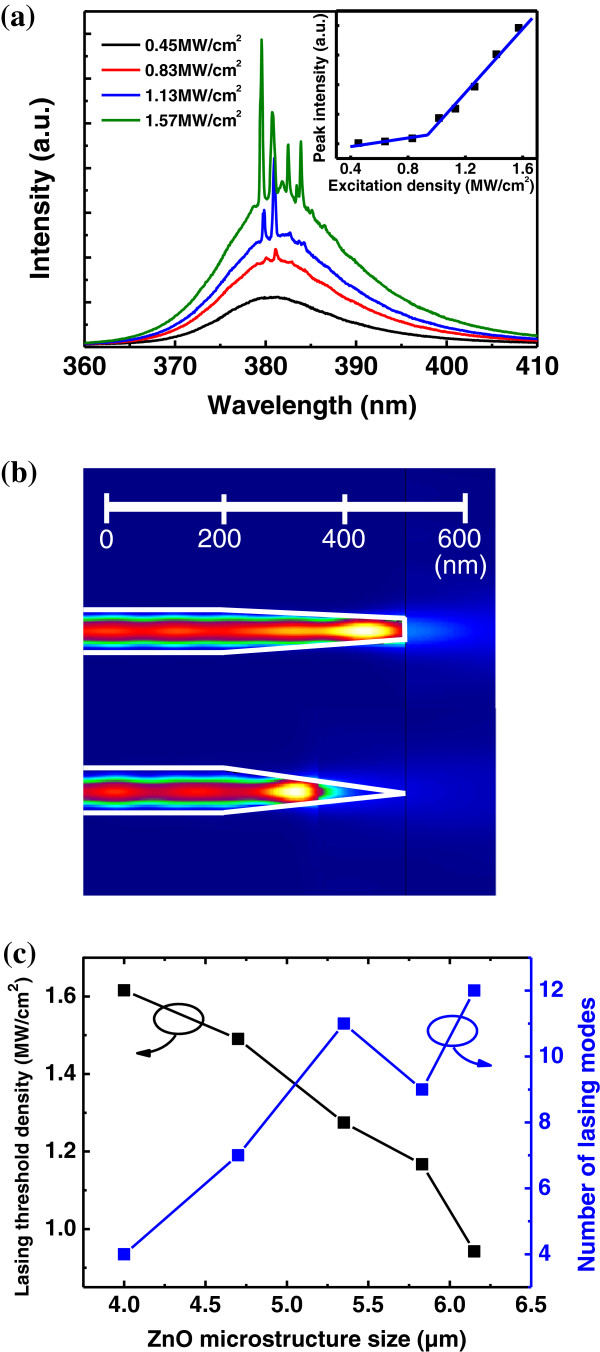
**Micro-PL spectra of individual ZnO microcavities, intensity profiles, and variation in the lasing threshold density. (a)** Micro-PL spectra of individual ZnO microcavities with the size of 6.15 μm upon different excitation densities of pulsed laser. The inset shows the plot of integrated PL intensity as a function of excitation density, exhibiting the lasing threshold of 0.94 MW/cm^2^. **(b)** Intensity profiles calculated for a flat-head tapered nanowire (top) and a highly tapered nanowire (bottom). **(c)** A plot of lasing threshold density versus individual ZnO microcavity size.

Next, we concentrated on the lasing characteristics of the individual urchin-like ZnO microstructures. Figure 4a shows a typical excitation-dependent μ-PL measurement of a ZnO microstructure with a size of 6.15 μm. The broad emission centered at 381 nm had no remarkable features at low excitation densities. As the excitation density increased, sharp peaks were observed at 379.5, 380.8, 382.5, and 383.8 nm. Furthermore, the peak intensities increased rapidly with further increases in the excitation density. The sharp PL emissions and nonlinear increase in the PL intensities with the excitation density indicated that lasing action was occurring, and the lasing threshold density was approximately 0.94 MW/cm^2^, as shown in the inset of Figure 4a. The width of the spectral line of the lasing peak was less than 0.15 nm. Therefore, the cavity mode had an intrinsically high quality (*Q*) factor, which was estimated to be 2,500 using the equation *Q* = *λ*/*δλ*, where *λ* is the peak wavelength. This *Q* factor was higher than those of other ZnO nano/microstructures [25,26]. The quality factor (*Q*) of the lasing spectra was estimated to be approximately 2,500, which was higher than that of our expectation.

For a conventional Fabry-Pérot (F-P) cavity, the *Q* factor can be expressed using the following equation [18]:

(1) $$Q=2\mathrm{\pi nD}/\lambda \left(1-R\right),$$

where *R* is the reflectivity of the two facets, *D* is the cavity length, *n* is the refraction index, and *λ* is the wavelength. *n* ~ 2.3 is the refractive index of ZnO, and *R* = (*n* − 1)^2^/(*n* + 1)^2^ = 0.16 is the reflectivity at the ZnO/air boundary. When the diameters were 10 and 0.5 μm, the corresponding *Q* factors were calculated to be 431 and 22, respectively. These values were much smaller than the above *Q* factor, which indicated that the lasing mechanism was not from the F-P cavity.

In the case of a whispering-gallery mode (WGM), the light was totally reflected by the six lateral sides of the ZnO nanowire at a 60° incident angle because the critical angle of the total internal reflection was approximately 25.8° at the ZnO/air boundary. However, the WGM was difficult to achieve because of the high loss (rough surface) and short gain length in an individual nanowire. Consequently, we excluded that the sharp spectral features were from a few high-quality nanowires.

To confirm the lasing mechanism of the ZnO microcavities, μ-PL measurements of different-sized individual microcavities were made. The PL spectra of different microcavities showed that the spacings between the adjacent sharp peaks were not the same when the sizes and morphologies of the microcavities were different. Therefore, we suggest that the lasing action used should be the RL action [27]. In the urchin-like ZnO microstructures, the body of the microstructures, functioning as an optical gain medium, can provide light amplification. By coherent scattering, the light forms multiple closed-loop optical paths that then serve as laser resonators. The lasing emission wavelength corresponds to the optical path loops in the microstructures. When the amplified light propagates from the body of the microstructure into tapered nanowires, a particular taper diameter is considered as a distributed mirror [28]. The amplified light cannot propagate to the taper, so it returns to the body of the microstructure, which results in efficient optical confinement and the recurrence of the amplified light in the urchin-like microstructure. The laser light eventually escapes through the rough surface of the body.

In order to reveal the light propagation in a ZnO tapered structure, the intensity distributions of electromagnetic fields were simulated using three-dimensional (3D) EigenMode Expansion methods (FIMMPROP), as shown in Figure 4b. Two types of nanotapered nanowires were selected: a highly tapered nanowire and a tapered nanowire with a flat head. We found that a greater fraction of the light was reflected and traveled back to the left inside the nanowire. Interestingly, the fraction of light transmission in the tapered structure with a flat head was greater than that in the highly tapered structure. In other words, the light confinement could be increased in the highly tapered structure. The simulation result indicated that our urchin-like microstructure with multiple-tapered nanowires could improve the light confinement and increase the possibility of light amplification, resulting in a higher *Q* factor for the urchin-like microstructures compared to other nano/microstructures.

Figure 4c shows the variation in the lasing threshold density as the size of the ZnO microcavities changed. Note that the larger-sized ZnO microcavities had a lower lasing threshold density than the smaller microcavities because the larger volume of the cavities increased the length of the optical gain. Thus, RL could be easily achieved. In addition, the number of resonance modes clearly increased as the size of the cavities increased. The number of lasing modes was also directly related to the size of the microcavities. Figure 4c also shows the number of lasing modes as a function of the size of the microcavities just above their lasing threshold. For the smallest microcavities, only four peaks were observed. As the size of the microcavities increased further, the number of lasing modes increased. The finite size of the cavities limited the number of lasing modes as a result of the gain competition between the random lasing microcavities. If the path loop of the cavity mode spatially overlapped other cavity loops, the lasing behavior did not occur. These results were in agreement with the theoretical calculation for RL [29].

## Conclusions

In conclusion, we reported a simple method for preparing urchin-like ZnO microlaser cavities via the oxidization of metallic Zn. The hexagonal Zn microcrystals were prepared using vapor-phase transport. After the oxidation of the Zn microcrystals, urchin-like ZnO microstructures were formed, and the mechanism of their crystal growth was proposed. For each individual urchin-like ZnO macrostructure, the laser presented a low threshold and high *Q* factor because the tapered nanowires could serve as effective optical reflectors to improve the optical confinement in the microstructures. The lasing characteristics such as the lasing mode and threshold were investigated. The results are significant for designing architectural nanotapered structures for advanced light management in other optoelectronic devices.

## Competing interests

The authors declare that they have no competing interests.

## Authors’ contributions

CHL and TYC synthesized the ZnO microstructures, carried out the structural characterization and PL measurements, and participated in the data interpretation. YFC and SYT were responsible for calculations of the electric field distribution and participated in the data interpretation. HCH initiated the study, designed all the experiments, analyzed the data, and prepared the manuscript. All authors read and approved the final version of the manuscript.
